# RETINAL VASCULITIS SEVERITY ASSESSMENT: Intraobserver and Interobserver Reliability of a New Scheme for Grading Wide-Field Fluorescein Angiograms in Retinal Vasculitis

**DOI:** 10.1097/IAE.0000000000003838

**Published:** 2023-05-23

**Authors:** Dhanach Dhirachaikulpanich, Savita Madhusudhan, David Parry, Salma Babiker, Yalin Zheng, Nicholas A.V. Beare

**Affiliations:** * Department of Eye & Vision Sciences, University of Liverpool, Liverpool, United Kingdom;; † Faculty of Medicine, Siriraj Hospital, Mahidol University, Bangkok, Thailand;; ‡ St Paul's Eye Unit, Liverpool University Hospitals NHS Foundation Trust, Liverpool, United Kingdom; and; § Liverpool Centre for Cardiovascular Science, University of Liverpool and Liverpool Heart & Chest Hospital, Liverpool, United Kingdom.

**Keywords:** cystoid macular edema, fluorescein angiography, optic disk leakage, posterior uveitis, retinal vascular leakage, retinal vascular occlusion, retinal vasculitis, visual acuity

## Abstract

**Purpose:**

Wide-field fluorescein angiography is commonly used to assess retinal vasculitis (RV), which manifests as vascular leakage and occlusion. Currently, there is no standard grading scheme for RV severity. The authors propose a novel RV grading scheme and assess its reliability and reproducibility.

**Methods:**

A grading scheme was developed to assess both leakage and occlusion in RV. Wide-field fluorescein angiography images from 50 patients with RV were graded by four graders, and one grader graded them twice. An intraclass correlation coefficient (ICC) was used to determine intraobserver–interobserver reliability. Generalized linear models were calculated to associate the scoring with visual acuity.

**Results:**

Repeated grading by the same grader showed good intraobserver reliability for both leakage (ICC = 0.85, 95% CI 0.78–0.89) and occlusion (ICC = 0.82, 95% CI 0.75–0.88) scores. Interobserver reliability among four independent graders showed good agreement for both leakage (ICC = 0.66, 95% CI 0.49–0.77) and occlusion (ICC = 0.75, 95% CI 0.68–0.81) scores. An increasing leakage score was significantly associated with worse concurrent visual acuity (generalized linear models, β = 0.090, *P* < 0.01) and at 1-year follow-up (generalized linear models, β = 0.063, *P* < 0.01).

**Conclusion:**

The proposed grading scheme for RV has good to excellent intraobserver and interobserver reliability across a range of graders. The leakage score related to present and future visual acuity.

## Abstract 2

Supplemental Digital Content is Available in the Text.

Retinal vasculitis is assessed by fluorescein angiography, but there is no agreed-on scheme for grading its severity in clinical practice/trials. The authors propose a new straightforward grading scheme using wide-field fluorescein angiography. The scheme demonstrates good to excellent interobserver and intraobserver reliability and a correlation between its severity score and vision.

Retinal vasculitis (RV) is an inflammation of the retinal vessels,^1^ which constitute the inner blood–retinal barrier. It has multiple causes, including infection, autoimmune diseases, inflammation, and neoplastic disorders, and can be idiopathic.^2,3^ Retinal vasculitis potentially leads to visual loss because of macular edema and vascular occlusion.

Assessing RV can be challenging. Fundoscopic findings include perivascular hemorrhage, perivascular infiltrate, vessel sheathing, vessel caliber abnormalities, nonperfused retina, and retinal neovascularization^4^ but can be subtle or absent when RV is subclinical. Currently, fluorescein angiography, preferably wide field, is the standard method to assess RV.^5^ Retinal vasculitis manifests as vascular leakage, occlusion of vessels, or vessel wall staining on the angiogram.^6^ Leakage or breakdown of the inner blood–retinal barrier can affect arterioles, venules, or capillaries. It is generally accepted that vascular leakage, either segmental or widespread,^7^ is an indicator of inflammation and disease activity.^8^ Inflamed vessels, either capillary or larger vessels, can occlude, resulting in retinal nonperfusion, and if extensive lead to neovascularization. Although an important indicator of severity, the presence of occlusion or nonperfusion is not a reliable indicator of active disease because it persists after inflammation has resolved.

Current clinical practice lacks agreement on a suitable severity classification or grading system for RV. Recent clinical trials have merely used the presence or absence of “active RV” as an endpoint, ignoring all information on severity.^9–11^ The Standardization of Uveitis Nomenclature working group announced in 2005 that “there was consensus that the definition of RV required more work.”^12^ Later, the Angiography Scoring for Uveitis Working Group (ASUWOG) developed a 40-point scoring scheme to assess fluorescein angiography for posterior uveitis in standard images.^13^ The ASUWOG scheme has not been widely adopted despite showing high interobserver reliability even in wide-field fluorescein angiography (WFFA) images.^14^ It has only been used in two published studies of Behcet uveitis^8^ including monitoring response to adalimumab.^15^ The ASUWOG scoring scheme is too detailed for practical clinical use or even for clinical trials.

In this study, we aimed to develop a grading scheme for RV that was meaningful and easy to use. We decided to focus on vascular leakage and occlusion as the main criteria for WFFA imaging and their extent in the peripheral or central retinal zones. We tested the reliability of our novel grading scheme by analyzing agreement between same and different graders and correlated severity scores with visual acuity.

## Methods

### Study Design

This was a single-center, retrospective clinical study of patients diagnosed with RV and treated at St Paul's Eye Unit, Liverpool University Hospitals NHS Trust, Liverpool, United Kingdom, between 2009 and 2022. The study was conducted in accordance with the Declaration of Helsinki and was approved by the NHS Research Ethics Committee of the North of Scotland (reference number 19/NS/0186).

Two investigators identified patients attending St Paul's Eye Unit with RV based on electronic medical records. The diagnosis of uveitis was based on the Standardization of Uveitis Nomenclature criteria.^12^ Eligible patients were older than 18 years with known RV in one or both eyes and had previous WFFA. Patients with proliferative diabetic retinopathy, retinal vascular disease other than RV, or other ocular diseases, which would interfere with WFFA assessment, were excluded. Informed consent was obtained from each patient.

Demographic data collected included age, sex, and race. We also collected the final etiological diagnosis of uveitis and the details of systemic immunosuppressant and local (ocular and periocular) treatment. Visual acuity was gathered as corrected visual acuity at the time of imaging and at 1-year follow-up. The corrected visual acuity was converted from Snellen to logarithm of the minimum angle of resolution for statistical analysis.

### Wide-Field Fluorescein Angiography

Retinal angiography was performed using SPECTRALIS HRA (Heidelberg Engineering, Heidelberg, Germany) (105° field of view) or HRA2 Scanning Laser Ophthalmoscope (Heidelberg Engineering, Heidelberg) with the use of Staurenghi Scanning Laser Ophthalmoscope 230 contact lens (Ocular Instruments, Inc, Bellevue, WI) (150° field of view)^7^ or Optomap P200DTX (200° field of view) or P200MA (200° field of view) (Optos, Dunfermline, United Kingdom).^16^ Angiograms were centered on the posterior pole of the fundus.

### New Retinal Vasculitis Clinical Grading for Wide-Field Fluorescein Angiography Images

Our grading scheme was based on graded scoring for the degree of leakage and occlusion on WFFA per eye of a patient. First, the quality of images was assessed for focus and clarity and any obscuration from media opacity and graded as ungradable (0), where it was not possible to assess leakage from the retinal vessels or retinal perfusion with confidence, or when less than two retinal quadrants were seen (see below). For gradable images, image quality was noted as poor (1), fair (2), or good (3).

**Fig. 1.**
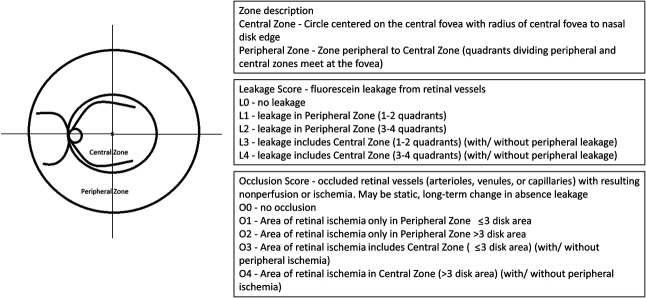
Newly proposed RV grading scheme.

We divided the fundus into peripheral and central zones. The central zone was defined by a circle centered on the foveal center with distance from the central fovea to the nasal optic disk edge as its radius. This zone was chosen to include all of the macula, the optic disk, and the temporal vascular arcades, all of which are critical to maintaining central vision. Vascular leakage or occlusion in this zone is more likely to have consequences for vision and is therefore deemed more severe than changes in the peripheral zone. All of the visible retina external to the central zone was defined as the peripheral zone. Both zones were divided into four quadrants based on horizontal and vertical lines intersecting at the fovea (Figure 1).

Retinal vascular leakage or “leakage” was defined as progressive hyperfluorescence coming from retinal vessels, either segments or full lengths of vessels.^13,17^ Leakage is usually evident early in the fluorescein imaging and progresses to cover a larger area around vessels in later shots. Staining, defined as vessel wall hyperfluorescence evident from mid-run onward without expansion,^17^ was not included because it is deemed not indicative of active disease. It did not include leakage at the level of the retinal pigment epithelium, which forms the outer blood–retinal barrier, leakage from optic disk, new vessels, or hyperfluorescence due to fluid-filled cysts in macular edema alone.

The leakage score was based on the number of quadrants showing the presence of leakage and their zone. Leakage in the peripheral zone was graded as L1, when 1 to 2 quadrants were involved, or L2, when 3 to 4 quadrants were involved. Leakage in the central zone was graded as L3 (1–2 quadrants) or L4 (3–4 quadrants) regardless of leakage in the peripheral zone. Thus, a leakage score of increasing severity from L0 to L4 was derived.

**Fig. 2.**
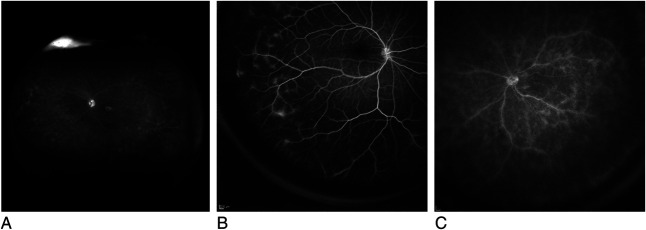
Example images for the leakage score: (**A**) leakage from RPE L0 (not identified as RV leakage), (**B**) leakage from vessels in peripheral zone L2 (identified as RV leakage), and (**C**) leakage from vessels in central zone L4 (identified as RV leakage).

**Fig. 3.**
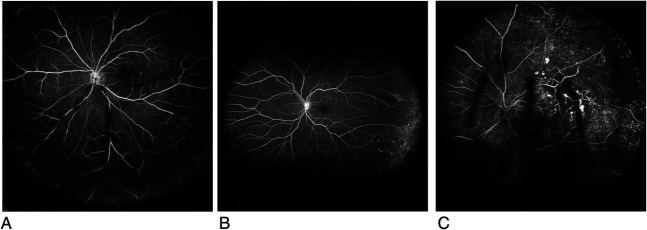
Example images for the occlusion score: (**A**) no occlusion O0, (**B**) occlusion in peripheral zone O2, and (**C**) occlusion in central zone O4.

The occlusion score was based on the presence of occluded retinal vessels resulting in nonperfusion or ischemia, the zone affected, and the area of ischemia. Occlusion in the peripheral zone was graded as O1, when nonperfusion covered an area of less than three disk areas, or O2, when more than three disk areas. Nonperfusion in the central zone was graded as O3, when less than three disk areas, or O4, when more than three disk areas. The cutoff of three disk areas was derived from the ASUWOG grading scheme. All the grading criteria are summarized in Figure 1. Figures 2,3 and **Supplemental Digital Contents 1** and **2** (see **Images**, http://links.lww.com/IAE/B981, http://links.lww.com/IAE/B982) show examples of grading the WFFA for RV using the proposed new scheme.

In addition, we included further questions on the predominant vessel type involved, the presence of cystoid macular edema (CMO) (on WFFA alone), and the presence of optic disk leakage (Table 1). This was to explore the utility of these assessments.

**Table 1.** Characteristics of 50 Recruited Patients

	No. of Patients (N = 50) (%)		No. of Eyes (N = 99) (%)		
			RV		Fischer's Exact
			(+) (N = 87)	(−) (N = 12)	*P* < 0.05
Gender		Type			
Female	29 (58%)	Intermediate uveitis with/without RV	22 (23.2%)	1 (8.3%)	No
Ethnicity		Only posterior uveitis	54 (57.6%)	3 (25%)	Yes
White British	38 (76%)	Panuveitis	7 (8%)	0 (0%)	No
Black or Black British African	3 (6%)	Clinical features			
Others	9 (18%)	Cataract	13 (14.9%)	2 (16.7%)	No
Age at diagnosis	Mean = 46.4 (IQR = 36.8–59)	Pseudophakia	5 (5.7%)	1 (8.3%)	No
Bilateral	38 (76%)	Vitreous hemorrhage	4 (4.6%)	0 (0%)	No
Diagnosis		Optic neuropathy	3 (3.4%)	0 (0%)	No
Idiopathic	24 (48%)	Epiretinal membrane	7 (8%)	0 (0%)	No
Behcet uveitis	4 (8%)	Macular atrophy	2 (2%)	0 (0%)	No
Sarcoidosis	3 (6%)				
SLE	3 (6%)	LogMAR CVA (at the time of imaging)	Mean = 0.295 SD = 0.54	Mean = 0.058 SD = 0.15	*t*-test *P* = 0.002
Unspecified systemic vasculitis	3 (6%)	−0.1–0 (6/5–6/6)	22 (25.3%)	8 (66.7%)	
Multiple sclerosis	3 (6%)	0.1–0.2 (6/7.5–6/9)	30 (34.5%)	3 (25%)	
Birdshot chorioretinopathy	2 (4%)	0.3–0.6 (6/12–6/24)	22 (25.3%)	1 (8.3%)	
Tuberculosis	2 (4%)	≥0.8 (≤6/36)	13 (14.9%)	0 (0%)	
Crohn disease	1 (2%)	Anterior chamber cells			
Granulomatosis with polyangiitis	1 (2%)	0	63 (72.4%)	12 (75.8%)	No
IRVAN	1 (2%)	0.5+	11 (12.6%)	0 (0%)	
Psoriatic arthritis	1 (2%)	1+	5 (5.7%)	0 (0%)	
Rheumatoid arthritis	1 (2%)	2+	2 (2.3%)	0 (0%)	
Vogt–Koyanagi–Harada disease	1 (2%)	3+	2 (2.3%)	0 (0%)	
Systemic treatment (at the time of imaging)		No data	4 (4.6%)	0 (0%)	
No systemic steroid	23 (46%)	Vitreous haze			No
Prednisolone <10 mg/day	6 (12%)	0	56 (64.4%)	12 (100%)	
Prednisolone ≥10, ≤20 mg/day	8 (16%)	0.5+	6 (6.9%)	0 (0%)	
Prednisolone >20, ≤40 mg/day	7 (14%)	1+	14 (16.1%)	0 (0%)	
Prednisolone >40 mg/day	6 (12%)	2+	4 (4.6%)	0 (0%)	
MMF	7 (14%)	3+	3 (3.4%)	0 (0%)	
Azathioprine	2 (4%)	No data	4 (4%)	0 (0%)	
Cyclophosphamide	2 (4%)	Vitreous cells			No
Tacrolimus	1 (2%)	Absent	50 (57.5%)	11 (91.7%)	
Methotrexate	1 (2%)	Present	33 (34.3%)	1 (8.3%)	
Adalimumab	2 (4%)	No data	4 (4%)	0 (0%)	
Rituximab	1 (2%)	Local treatment			
		Topical steroid	23 (26.4%)	1 (8.3%)	No
		Intravitreal steroid (any previous)	3 (3.4%)	1 (8.3%)	No
		Vitrectomy	2 (2.2%)	0 (0%)	No
		Photocoagulation	5 (5.7%)	0 (0%)	No

### Evaluation of the Grading Scheme

One investigator selected two images from each eye and masked the clinical data and shuffled the participant order for intraobserver testing. This investigator was excluded from the grading exercise. The graders were two uveitis specialists (Consultants 1 and 2), a trained senior ophthalmic image grader, and an ophthalmology higher specialist trainee (resident). All the graders were trained by the same example image sets before grading and undertook a pilot exercise on scoring for images independently. For intraobserver reliability, the ophthalmic image grader was asked to grade the same image set again after a gap of 1 month. For the purpose of analyzing interobserver reliability, the first grading of the ophthalmic image grader was used. The grading outcomes of Consultant 1 were used for subsequent analysis.

### Statistical Analysis

All analyses were performed using SPSS 22.0 (SPSS Inc, Chicago, IL). The data are shown as mean, percentage, and SD. Student *t*-tests or chi-square tests were applied to compare the variables. For intraobserver and interobserver agreement, we calculated the intraclass correlation coefficient (ICC) for ordinal data (leakage score, occlusion score, and quality of image) and Fleiss' kappa statistics for categorical data (presence of cystoid macular edema, presence of optic disk leakage, and predominant vessel type involved). A single-measurement, absolute agreement with a two-way mixed effects model was applied for the ICC. The interpretation of both ICC and Fleiss' kappa was as follows: less than 0.40 as poor, 0.40 to 0.59 as fair, 0.60 to 0.74 as good, and 0.75 to 1.00 as excellent.^18^

Univariable and multivariable generalized linear regression analyses were used to calculate the association of the new grading scheme with the corrected visual acuity. The generalized estimating equations accounted for the elimination of the intereye correlation. Factors showing a *P* value less than 0.1 in univariable analysis were consequently analyzed in the multivariable analysis. A *P* value less than 0.05 was considered statistically significant for all analyses.

## Results

A total of 99 eyes from 50 patients with RV were recruited for this study. Table 1 summarizes the patients' characteristics and diagnoses. Of the 50 patients, 29 (58%) were female, most were White British (38, 76%), and the mean age at diagnosis was 46.4 years (interquartile range = 36.8–59). Thirty-eight (76%) patients had bilateral RV. Of 99 eyes, there were 87 eyes with RV and 12 without. The most common presenting symptom of the eyes with RV was reduced/blurred vision (49 with RV and 1 without RV, *P* < 0.05). The anatomical type of uveitis from 87 RV eyes included 22 (23.2%) intermediate uveitis, 54 (57.6%) posterior uveitis, and 7 (8%) panuveitis. Other associated ophthalmologic complications in eyes with RV were cataracts (13, 14.9%), pseudophakia (5, 5.7%), vitreous hemorrhage (4, 4.6%), optic neuropathy (3, 3.4%), epiretinal membrane (7, 8%), and macular atrophy (2, 2%). At the time of WFFA imaging, the mean logarithm of the minimum angle of resolution corrected visual acuity of eyes with RV was 0.295 and 0.058 in eyes without RV (*t*-test, *P* value < 0.05). Local treatment received in eyes with RV was topical steroid (23, 26.4%), intravitreal steroid (3, 3.4%), vitrectomy (2, 2.2%), and photocoagulation (5, 5.7%). From 50 patients, 27 (54%) received systemic treatment with oral prednisolone, 7 (14%) oral mycophenolate mofetil, 2 (4%) oral azathioprine, 2 (4%) intravenous cyclophosphamide, 1 (2%) oral tacrolimus, 1 (2%) oral methotrexate, 2 (4%) subcutaneous adalimumab, and 1 (2%) intravenous rituximab.

### Intraobserver and Interobserver Reliability

Intraobserver reliability showed excellent agreement for leakage (ICC 0.845, 95% CI 0.777–0.893) and good agreement for occlusion (ICC 0.824, 95% CI 0.747–0.878). For additional questions, the intraobserver reliability indicated fair agreement for CMO (kappa 0.579, 95% CI 0.573–0.586), fair agreement for optic disk leakage (kappa 0.509, 95% CI 0.503–0.575), excellent agreement for the quality of imaging (ICC 0.822, 95% CI 0.742–0.879), and fair agreement for the predominant vessel type involved (kappa 0.465, 95% CI 0.461–0.468).

The interobserver reliability was investigated for four graders scoring independently. The result revealed good agreement for the leakage score (ICC 0.655, 95% CI 0.491–0.769) and excellent agreement for the occlusion score (ICC 0.750, 95% CI 0.679–0.813). It is noted that the ICC between Consultants 1 and 2 has the highest agreement for the leakage (ICC 0.795, excellent).

There were imbalances in the score distribution. For instance, using Consultant 1 as ground truth, the commonest scores for leakage were L4 (37/96, 38.5%) and L0 (13/96, 13.5%). The raw agreement between Consultants 1 and 2 was higher for L0 and L4 than L1–L3. As expected, there was more leakage than occlusion, and O0 was the commonest occlusion score (64, 66.7%).

For additional questions assessing WFFA, the intraobserver reliability showed good agreement for CMO (kappa 0.638, 95% CI 0.635–0.640), poor agreement for optic disk leakage (kappa 0.293, 95% CI 0.291–0.296), fair agreement for the quality of WFFA imaging (kappa 0.565, 95% CI 0.449–0.609), and poor agreement for the predominant vessel type involved (kappa 0.266, 95% CI 0.265–0.267). The results of interobserver reliability are summarized in Table 2. As assessing CMO using WFFA is not standard practice, we additionally reviewed macular optical coherence tomography (OCT) scans acquired within 2 weeks (before or after) of WFFA in the study eyes for the presence/absence of CMO. Macular OCT scans were available in 82 study eyes. The result indicated fair agreement between assessing CMO by WFFA and OCT (kappa 0.444, 95% CI 0.317–0.571).

**Table 2.** Intraobserver and Interobserver Reliability of RV Grading From Four Independent Graders

	Leakage Score (ICC)	Occlusion Score (ICC)	CMO (Kappa)	Optic Disk Leakage (Kappa)	Quality of WFFA Imaging (ICC)	Predominant Vessels (Kappa)
Intraobserver reliability	0.845 (95% CI 0.777–0.893)	0.824 (95% CI 0.747–0.878)	0.579 (95% CI 0.573–0.586)	0.509 (95% CI 0.503–0.575)	0.822 (95% CI 0.742–0.879)	0.465 (95% CI 0.461–0.468)
Interobserver reliability						
Consultant 1–Consultant 2	0.795	0.741	0.836	0.276	0.498	0.424
Consultant 1–ophthalmic grader	0.750	0.720	0.426	0.477	0.467	0.258
Consultant 1–resident	0.525	0.856	0.779	0.497	0.570	0.226
Consultant 2–ophthalmic grader	0.755	0.641	0.376	0.372	0.555	0.192
Consultant 2–resident	0.519	0.771	0.765	−0.191	0.617	0.298
Ophthalmic grader–resident	0.574	0.770	0.554	0.092	0.702	0.133
All (4 graders)	0.655 (95% CI 0.491–0.769)	0.750 (95% CI 0.679–0.813)	0.638 (95% CI 0.635–0.640)	0.293 (95% CI 0.291–0.296)	0.565 (95% CI 0.449–0.669)	0.266 (95% CI 0.265–0.267)

### Association of Retinal Vasculitis Scoring With Visual Acuity

**Fig. 4.**
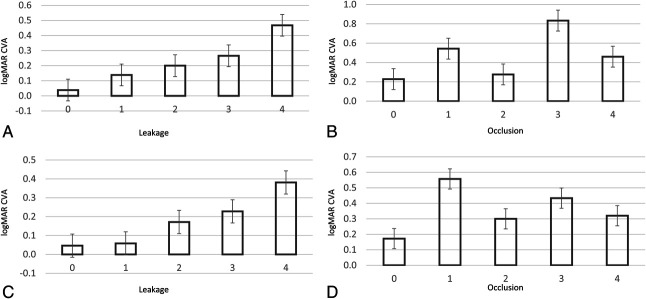
Graph showing the relation between clinical grading and visual acuity: (**A**) logMAR CVA at date image obtained and leakage score, (**B**) logMAR CVA at date image obtained and occlusion score, (**C**) logMAR CVA at 1-year follow-up and leakage score, and (**D**) logMAR CVA at 1-year follow-up and occlusion score. The error bars show the standard error.

We compared the RV scoring with visual acuity using generalized linear regression analyses with generalized estimating equations (Table 3, Figure 4). The univariable analysis showed that worse visual acuity was associated with higher leakage scores (β = 0.105, *P* = 0.001) as well as older age (β = 0.005, *P* = 0.04), male sex (β = 0.162, *P* < 0.05), and presence of optic disk leakage (β = 0.202, *P* = 0.027). The multivariable analysis showed only higher leakage score (β = 0.090, *P* = 0.008) and older age (β = 0.006, *P* = 0.016) as statistically significant associations with visual acuity.

**Table 3.** Association Between Clinical Variables Includes RV Grading With Visual Acuity Using a Generalized Linear Model With a Generalized Estimating Equation

Variables	Date of Imaging						1-Year Follow-up					
	Univariable			Multivariable			Univariable			Multivariable		
	Standardized Coefficient β		*P*	Standardized Coefficient β		*P*	Standardized Coefficient β		*P*	Standardized Coefficient β		*P*
	Value	95% CI		Value	95% CI		Value	95% CI		Value	95% CI	
Age (years)	0.005	0.00024–0.011	0.04	0.006	0.001–0.011	0.016	0.005	0.00049–0.0092	0.029	0.005	0.002–0.009	0.004
Gender (females)	−0.162	−0.323–0.000311	0.0495	−0.111	−0.278–0.056	0.192	−0.066	−0.246–0.114	0.474			
Dose of prednisolone	0.004	−0.0003–0.009	0.067	0.003	−0.002–0.008	0.290	0.02	−0.002–0.005	0.360			
No. of immunosuppressants	−0.006	−0.085–0.073	0.879				−0.015	−0.116–0.086	0.765			
Leakage score	0.105	0.054–0.156	0.0001	0.090	0.023–0.158	0.008	0.089	0.037–0.141	0.001	0.063	0.018–0.107	0.006
Occlusion score	0.073	−0.002–0.148	0.056	0.048	−0.024–0.121	0.190	0.059	−0.005–0.123	0.071	0.050	−0.007–0.106	0.083
CMO	0.169	−0.008–0.346	0.061	−0.041	−0.269–0.186	0.721	0.1	−0.079–0.278	0.273			
Optic disk leakage	0.202	0.022–0.381	0.027	0.144	−0.049–0.336	0.143	0.241	0.022–0.46	0.031	0.233	0.048–0.418	0.014

At 1-year follow-up, the univariable analysis showed that higher leakage score (β = 0.089, *P* = 0.001), higher age (β = 0.005, *P* = 0.029), and presence of optic disk leakage (β = 0.241, *P* = 0.03) were associated with worse visual acuity. The multivariable analysis showed higher leakage score (β = 0.063, *P* = 0.006), older age (β = 0.005, *P* = 0.004), and optic disk leakage (β = 0.233, *P* = 0.014) as a statistically significant association with visual acuity.

## Discussion

There is currently no accepted grading scheme for RV, resulting in unquantified clinical assessment, poor communication between clinicians, and only a binary outcome for clinical trials in uveitis (present or absent) meaning much lost data. We present a pragmatic grading scheme based on WFFA, which is not time consuming and encompasses the important concepts of vascular leakage (activity) and occlusion (damage). We demonstrate that it has intraobserver and interobserver reliability across different cadres of observers and relates to function in the form of visual acuity.

At present, there are no Standardization of Uveitis Nomenclature criteria for assessing the severity of RV.^12^ The ASUWOG scoring system^13^ has been developed but has 40 points of assessment and is not practical for use clinically or in clinical trials. It has not been widely taken up and has only been tested in Behcet disease.

The recent decades have made significant progress in noninvasive imaging methods, specifically OCT and OCT-A, but these are not yet suitable alternatives to using WFFA for the assessment of RV,^19,20^ which remains the standard method for diagnosis and severity assessment.^6^ In this current study, we emphasize two major angiographic features of RV, leakage and occlusion, and we propose grading their involvement within a peripheral zone and a central zone on a scale of severity. Our investigation of intraobserver and interobserver agreement indicates that this grading scheme has good or excellent concordance, which means that it is reliable and reproducible between observers. Our interobserver agreement in the leakage score has a higher agreement between two uveitis consultants rather than an ophthalmic grader or trainee. This suggests that the accuracy of the assessment improves with clinical experience, and additional training may improve less experienced cadres.

Both univariable and multivariable analyses found that the leakage but not occlusion score was associated with visual acuity. The relationship between leakage and vision is meaningful in that it is measuring disease activity with an impact on visual function. Severity of vascular leakage thus may potentially serve as a clinical biomarker, indicating the need for more intensive treatment.^21,22^ Our current analysis did not show an association between occlusion grade and visual acuity. Occlusion will only directly affect visual acuity if it affects the fovea, which will result in severe loss, so there is not an expected direct relationship with generalized severity. However, greater areas of occlusion and involvement of the posterior pole rather than the periphery have a greater chance of having a devastating impact on acuity. This is supported by previous research, which indicated a relationship between the foveal avascular zone size but not peripheral ischemia with poor visual outcome^5^ in eyes with RV secondary to uveitis.

We found poor interobserver agreement for optic disk leakage. This feature may be related to overall uveitis activity or specific optic nerve involvement and RV. We suggest that it not be included in the grading of RV severity. We also assessed the concordance of observers to identify the predominant type of vessel involved in inflammation, which is said to aid the diagnosis of the underlying disease. Unfortunately, this only had poor agreement, and we suggest that this should not be included in a RV severity grading scale.

Besides clinician grading, automated computerized algorithms developed to grade retinal imaging are promising in many retinal diseases.^23^ Automated algorithms could quantify areas affected by leakage and occlusion on continuous scales with weighting for central versus peripheral involvement. Attempts at automated grading of retinal vascular leakage, including patients with diabetic retinopathy^24^ and patients with malarial retinopathy,^25,26^ have given promising results. However, an automated computerized method incorporating both leakage and occlusion, specific to patients with RV, is still desirable.

This study was designed to retrospectively use images obtained in routine clinical practice from patients presenting with RV to a tertiary uveitis center. It was limited by using observers all from one center, albeit from different cadres. There were more examples of leakage than occlusion, as is encountered in clinical practice. The images used were not equally distributed in terms of scores for leakage and occlusion; in particular, there were more L4 images than L1, L2, or L3. This is in part a result of the simplicity of the scheme and also due to the limited sample size, but the relationship of the leakage grade to visual acuity supports this division of grades. Visual acuities were uniformly collected using Snellen charts and with the patients' own correction or pinhole, rather than refracted (best-corrected) visual acuity.

In conclusion, we have proposed a new severity grading scheme for RV focused on leakage and occlusion on retinal angiography and found it to have suitable intraobserver and interobserver reliability. The leakage score relates to visual acuity, which supports its use as a biomarker for disease activity. Prospective studies could further validate this scoring scheme for RV and how it relates to treatment decisions and outcomes.

## Supplementary Material
