# Supplementary figures and images for: RETINAL VASCULITIS SEVERITY ASSESSMENT: Intraobserver and Interobserver Reliability of a New Scheme for Grading Wide-Field Fluorescein Angiograms in Retinal Vasculitis
Source: Retina. 2023 May 23;43(9):1534–43. doi: 10.1097/IAE.0000000000003838 (PMC10442128; doi:10.1097/IAE.0000000000003838)

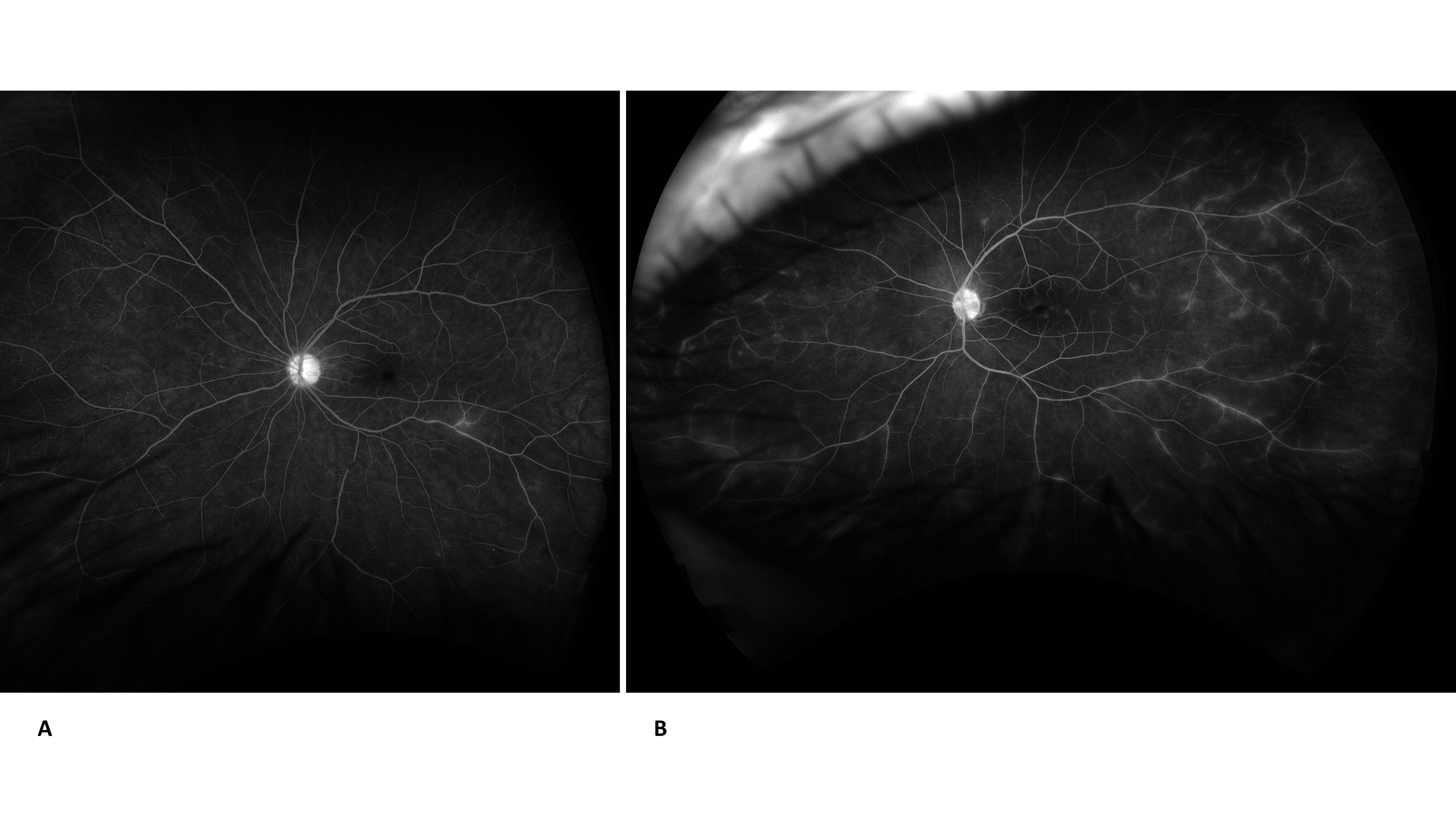

Supplement: Supplementary file 1 [file retina-43-1534-s001.tif]

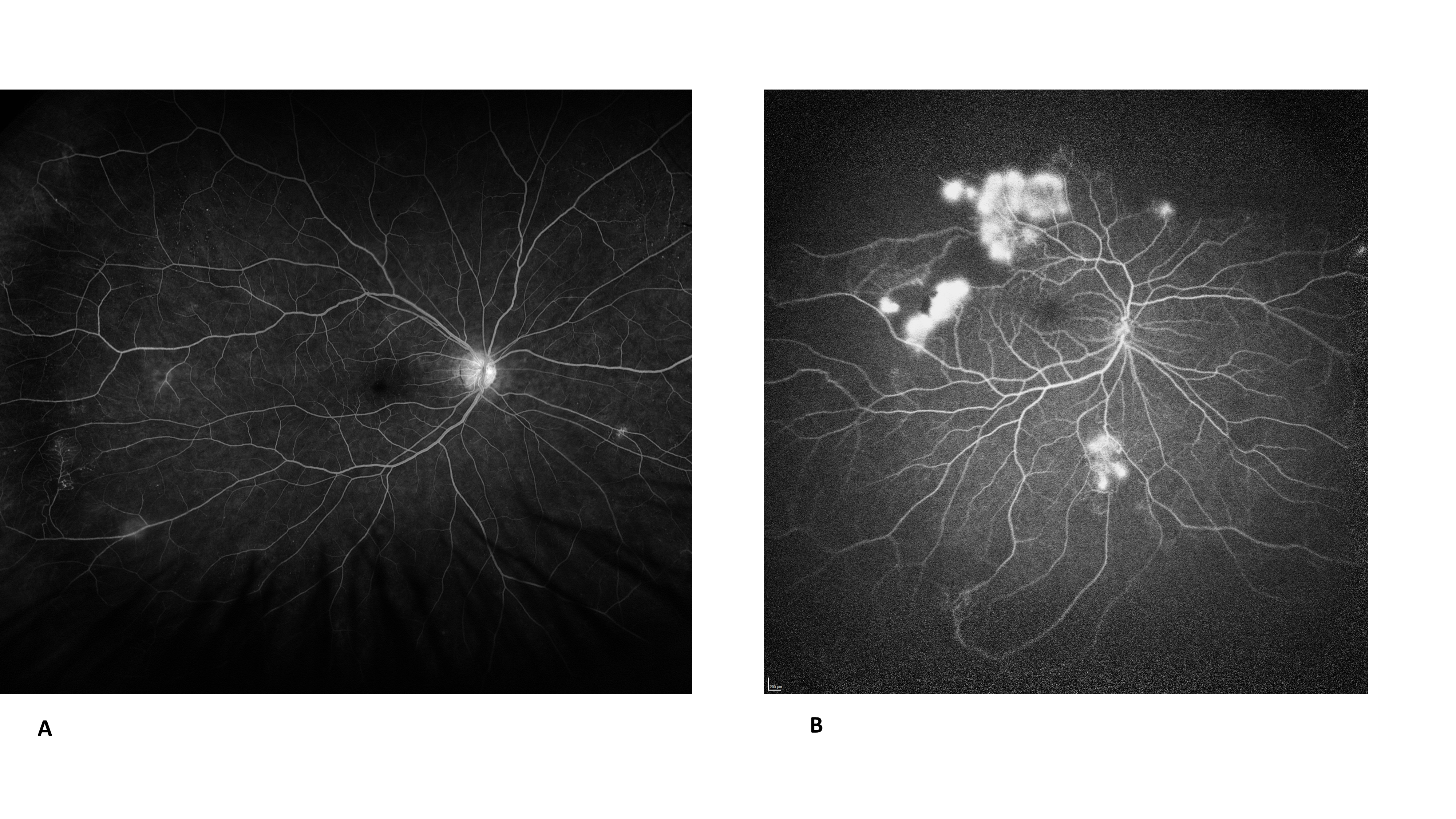

Supplement: Supplementary file 2 [file retina-43-1534-s002.tif]
